# Direct and indirect effects of land use on microbiomes of trap-nesting solitary bee larvae and nests

**DOI:** 10.3389/fmicb.2024.1513096

**Published:** 2025-01-09

**Authors:** Birte Peters, Sara Diana Leonhardt, Michael Schloter, Alexander Keller

**Affiliations:** ^1^Department for Animal Ecology and Tropical Biology, Biocenter, University of Würzburg, Würzburg, Germany; ^2^Center for Computational and Theoretical Biology, University of Würzburg, Würzburg, Germany; ^3^Department of Biodiversity and People, Helmholtz Center Leipzig, German Centre for Integrative Biodiversity Research (iDiv), Leipzig, Germany; ^4^Plant-Insect Interactions, TUM School of Life Science Systems, Technical University of Munich, Freising, Germany; ^5^Comparative Microbiome Analysis, Helmholtz Centrum Munich, Munich, Germany; ^6^Cellular and Organismic Networks, Faculty of Biology, Ludwig-Maximilians-Universität Munich, Planegg-Martinsried, Germany

**Keywords:** solitary bee microbiome, metabarcoding, pollination, Biodiversity Exploratories, grasslands, *Osmia bicornis*

## Abstract

**Introduction:**

The global decline in biodiversity and insect populations highlights the urgent need to conserve ecosystem functions, such as plant pollination by solitary bees. Human activities, particularly agricultural intensification, pose significant threats to these essential services. Changes in land use alter resource and nest site availability, pesticide exposure and other factors impacting the richness, diversity, and health of solitary bee species. In this study, we investigated yet another facet currently less well investigated in such context: Microbial communities associated with wild bees play crucial roles in larval development, metabolism, immunity and overall bee health. However, the drivers and dynamics of healthy microbiome in solitary bees are still poorly understood, especially regarding the direct and indirect effects of land use on the diversity and composition of these microbial communities.

**Methods:**

We examined bacterial communities in the offspring and nest materials of the Megachilid trap-nesting solitary bee, Osmia bicornis, along a gradient of land use intensification by 16S rRNA gene metabarcoding. Given that landscape composition, climatic conditions, and food resources are known to influence microbial compositions in solitary bee species, we hypothesized that land use changes would alter resources available for food and nest material collection and thereby affecting the microbiomes in offspring and their nest environments. We anticipated reduced microbial diversity and altered composition with increased land use intensification, which is known to decrease the number and diversity of resources, including the pool of floral and soil bacteria in the surrounding environment.

**Results:**

As expected, we observed significant shifts in the bacterial composition and diversity of bees and their nests across varying degrees of land use intensity, differing in management types and the availability of flowers. The Shannon diversity of bacteria in nest materials (larval pollen provision, soil nest enclosure) and larval guts decreased with increasing land use intensity. However, the pupae microbiome remained unaffected, indicating a reorganization of the microbiome during metamorphosis, which is not significantly influenced by land use and available resources.

**Discussion:**

Our findings provide new insights into the factors shaping environmental transmission and changes in solitary bee microbiomes. This understanding is crucial for comprehending the impacts of intensive land use on wild bee health and developing strategies to mitigate these effects.

## Introduction

Worldwide, the decline in biodiversity and insect populations raises concerns about the need to conserve valuable ecosystem functions, such as pollination of plants by solitary bees (Ricketts et al., 2016; van Klink et al., 2020; Wagner, 2020; Wagner et al., 2021). Prominent threats of such ecosystem services are anthropogenic activities, such as agricultural intensification (Potts et al., 2010; Murphy and Romanuk, 2014; Seibold et al., 2019). Changes in land use directly impact the availability, quality and diversity of (nesting and floral) resources, which in turn affect the richness, diversity and overall health of solitary bee species (Roulston and Cane, 2002; Michener, 2007; Roulston and Goodell, 2011; Roger et al., 2016; Westrich, 2019; Parreño et al., 2022; Peters et al., 2022). In addition to the specific floral requirements, such as pollen and nectar (Roulston and Cane, 2002; Tasei and Aupinel, 2008), the microbial communities associated with wild bees play an increasingly recognized role in larval development, metabolic functions and immunity (Engel et al., 2012; Lee et al., 2015; Leonhardt et al., 2022). This includes the microbial composition of larval guts, as well as of the nesting environment, which can either inhibit pathogen growth or serve as an external rumen (Keller et al., 2018; Dharampal et al., 2020; Voulgari-Kokota et al., 2020).

However, there is still limited understanding regarding the drivers, functions, acquisition, and changes in healthy microbiome of solitary bees, particularly during their metamorphosis (Graystock et al., 2017; Voulgari-Kokota et al., 2019b; Keller et al., 2021). The horizontal transmission of microbes from floral resources through the collection of pollen provisions is recognized as a significant pathway for microbiome acquisition (Dharampal et al., 2019; Voulgari-Kokota et al., 2019a; Steffan et al., 2024). In addition to pollen-associated microbiomes, other environmental bacteria originating from nesting resources, such as soils, are introduced into solitary bee nests, where they can proliferate (Rothman et al., 2019; Keller et al., 2021). Previous research on the alfalfa leaf-cutting bee highlighted that nesting components like leaf materials may additionally impact the microbial community within bee nests (Rothman et al., 2019; Cohen et al., 2020). Furthermore, trap-nesting Megachilid solitary bee species such as *Osmia bicornis* utilize soil to create distinct chambers within reed nests to protect solitary eggs (Danforth et al., 2019). Once sealed, individual nest chambers no longer receive active maternal care, preventing the continuous vertical transmission of bacteria between mothers, siblings, and offspring (Voulgari-Kokota et al., 2019b). This could potentially be advantageous, creating favorable conditions for the growth of beneficial bacteria (Hammer et al., 2023).

Moreover, landscape composition (Muñoz-Colmenero et al., 2020), climatic conditions (McFrederick and Rehan, 2019), natural surroundings, food resources and wild bee diversity in the ecosystem may all influence the microbial composition associated with and among different solitary bee species (Keller et al., 2021; Cohen et al., 2022; Shell and Rehan, 2022). Land use changes do not only affect the presence of solitary bee species, but have direct or indirect effects on their foraging patterns by altering the availability, diversity, quantity and quality of floral nutritional resources (Peters et al., 2022). Foraging preferences and the availability of flowers that act as microbial transmission hubs are supposed to influence the microbiome associated with solitary bees and pollen provision and expose them to a variety of bacteria which could contribute to successful larval development (Westreich et al., 2023). This includes potential beneficial and pathogenic bacteria, parasites and fungi (Keller et al., 2013; McFrederick et al., 2017; Zemenick et al., 2019; Keller et al., 2021; Dharampal et al., 2022; Weinhold et al., 2024). As land use also influences the composition of floral microbiomes associated with these resources (Gaube et al., 2020) as well as soil microbial communities (Schöps et al., 2018), landscapes with altered or reduced access to suitable foraging resources can lead to the dysbiosis of both floral and bee microbiomes or a reduction of bacteria (Morris et al., 2020; Christensen et al., 2021; Nguyen and Rehan, 2023). For instance, studies examining the effects of land use across urban land use gradients have found variations in bee microbial compositions, with an overrepresentation of beneficial plant associated bacteria in areas with low urban development (Nguyen and Rehan, 2022). However, it remains unclear how land use effects translate into the microbial composition of nest materials and offspring in solitary bees (Voulgari-Kokota et al., 2019b).

In this study, we investigated the nest microbiome of the Megachilid trap-nesting solitary bee, *Osmia bicornis*, using a metabarcoding approach based on the 16S rRNA gene. We conducted our field work along land use intensification gradients in managed grasslands across three different regions in Germany. Firstly, we examined whether there are differences in the bacterial communities present in various types of bee nest samples (larval pollen provisions, soil nest enclosures, larvae, and pupae). We hypothesized that bacterial communities changed over the bee’s development from larvae to pupae. Secondly, we tested whether the bacterial communities associated with bee larvae and pupae were influenced by the bacterial communities of the pollen provision and soil nest enclosures. We hypothesized that both larval pollen provision and soil nest enclosure serve as potential bacterial acquisition pathways, affecting the developmental stages of *O. bicornis* (bee larvae and pupae) and consequently shaping the bacterial compositions and community structures. Lastly, we investigated whether bacterial communities in different bee nest sample types were affected by land use intensification. We hypothesized that increased agricultural management intensity, characterized by more frequent or intense mowing, grazing, and fertilization, would result in lower bacterial diversity and altered composition due to the decreasing diversity of foraging resources and environmental bacterial pools.

## Materials and methods

### Area of sampling and sample type acquisition

The study was conducted in three geographically distinct regions in Germany: the UNESCO Biosphere Reserve area Schorfheide-Chorin (SCH) in the northeast, the National Park Hainich-Dün (HAI) in the center, and the UNESCO Biosphere Reserve Swabian Alb (ALB) in the south. We selected 27 grassland plots (9 per region) as part of the long-term Biodiversity Exploratories project (Fischer et al., 2010) (Supplementary Figure SM1). Each 50 m × 50 m plot represented different land use intensity types, including meadows, mowed pastures, grazed pastures, and fertilized and unfertilized areas, with management types extending beyond plot borders (Blüthgen et al., 2012) (Supplementary Table SM1). Detailed framework information is available in Fischer et al. (2010).

Bees were sampled according to legal requirements with permits ALB: AZ: 55-8/8848.02-07, HAI: AZ: 63.02/15.02.11-bio_expl2017.2 & AZ: 1011-17-301, SCH:AZ: 4743/128+5#69122/2018. We used vegetation records from BExIS public datasets (IDs 23,586 and 24,247: Vegetation Records for 150 Grassland EPs 2008–2018) to assess plot vegetation Shannon diversity (Schäfer et al., 2018; Bolliger et al., 2020), as well as data on management and land use intensity (LUI) from dataset IDs 25,086 and 31,514 (Lorenzen et al., 2023; Ostrowski et al., 2023). Land use intensity was categorized into low (LUI < 1.5), intermediate (LUI 1.1–2.3), and high (LUI > 2.3). Additionally, we used plant pollen dataset ID 27229 from our previous study on *O. bicornis* larval pollen provisions to analyze the effects of land use intensity on pollen plant diversity (Peters et al., 2022).

### Sampling of solitary bee nests

To collect various sample types (larval pollen provisions, soil nest enclosures, bee larvae and pupae) of Megachilid solitary bee nests, we used artificial perpendicular trap nests made of plastic tubes with 60–80 hollow reed sticks (length ~ 20 cm, width 4–12 mm). In early spring 2017, the trap nests were placed at the fence of a weather station in the center of each grassland plot. From March to July in 2017 and 2018, reed sticks were regularly checked for solitary bee occupation. Sticks with closed entrances were carefully removed and replaced with empty ones. The collected sticks were then transported to the laboratory (see Peters et al., 2022 for more details).

We classified solitary bee species based on reed nest closures and bee morphology, following the methodology outlined by Amiet, (2017). Reed cane internodes were opened, and solitary bee larvae, pupae, larval pollen provisions and soil nest enclosure materials were collected separately using sterile spatulas and forceps. We specifically focused on nests of *Osmia bicornis*, which was the only species present across all three bioregions and the entire LUI gradient. Well-developed larvae and pupae, as well as pollen provisions, soil nest enclosures from the reed cells were transferred into autoclaved tubes and a total of 144 samples immediately frozen at −20°C for preservation. Afterwards reeds were carefully re-closed to allow further larval development of remaining bees to facilitate more detailed classification after enclosure.

### Metabarcoding

Genomic DNA extraction for bacterial analysis the ZymoBIOMICS^™^ 96 DNA Kit (Zymo Research) was utilized following the manufacturer’s protocol. To generate a pooled amplicon library for the 16S rRNA V4 region, we employed a dual-indexing strategy following the methods described in Kozich et al. (2013) and Illumina (2017). To minimize amplification biases all PCRs were performed in triplicates and with a proofreading Phusion High-Fidelity PCR Master Mix with HF Buffer according to manufacturer’s instructions (Thermo Fisher Scientific, Waltham, United States). Negative controls, including (i) DNase/RNase Free Water (Zymo Research) and (ii) DNA/RNA Shield^™^ (Zymo Research) and a Microbial Community Standard (Zymo Research) as a positive control, were included for quality control purposes. All controls underwent the same workflow as the other samples. To prevent the amplification of chloroplast related sequences in pollen samples, pPNA blocking primer (PNA Bio Inc., Newbury Park, United States) were applied at a final concentration of 0.3 μM during the PCR reactions. Following the method described by Lundberg et al. (2013). PCR conditions were as follows: initial denaturation at 95°C for 4 min, followed by 30 cycles of 95°C for 40 s, annealing at 55°C for 30 s (including PNA clamping at 75°C for 10 s), extension at 72°C for 60 s and a final extension step at 72°C for 5 min. Triplicates were pooled per sample, checked by gel electrophoresis on 1.5% agarose gels for successful amplification and stored at 4°C. Samples were normalized in DNA amounts using the Invitrogen SequalPrep Plate Normalization Kit (Thermo Fisher Scientific) and purified with AMPure beads (Agilent, Santa Clara, United States). The normalized library was pooled and its fragment length distributions assessed using High Sensitivity DNA Chips on a Bioanalyzer 2200 (Agilent). The final library was quantified using the Qubit II Fluorometer with the dsDNA High-Sensitivity Assay Kit (Thermo Fisher Scientific), diluted to Illumina MiSeq requirements (Illumina, 2013, 2016, 2017), complemented with 5% of Illumina PhiXv3 and then loaded into a 500 cycle Illumina MiSeq cartridge following the manufacturer’s protocol (Illumina, 2013, 2017). Sequencing was performed on an Illumina MiSeq device (Illumina Inc., San Diego, United States) at the Department of Human Genetics of the University of Würzburg, Germany.

### Bioinformatics

For sequence analysis we utilized VSEARCH v.2.15.1 (Rognes et al., 2016) according to the pipeline available at https://github.com/chiras/metabarcoding_pipeline (Leonhardt et al., 2022). Forward and reverse reads were merged (with a minimum overlap of 10 bp), and the sequences were filtered based on length (>250 bp) and quality (E_max_ < 1, no ambiguous base pairs). Singleton reads were excluded, and *de-novo* chimera filtering was performed (Edgar and Flyvbjerg, 2015). Sequences were denoised and dereplicated into amplicon sequence variants (ASVs) using the Unoise3 algorithm (Edgar, 2016b). Taxonomy assignment of 16S rRNA gene sequences was conducted using the RDP v18 reference database using SINTAX with a threshold of 0.8 (Edgar, 2016a). Additionally, individual ASVs at the species level were double checked against GenBank (Zhang et al., 2000) using the NCBI BLASTn (Altschul et al., 1990). ASVs were filtered that showed conspicuous distributions in positive and negative controls, as well as sequences related to mitochondria or remaining chloroplasts. Samples with less than 1,000 reads were excluded from further analysis.

### Statistical analysis

Data was analyzed in *R* 4.0.2 (R core, 2017) using the packages *phyloseq v1.22.3* (McMurdie and Holmes, 2013), *vegan v2.5–2* (Oksanen et al., 2013), *lme4 v1.1–21* (Bates et al., 2015), *multcomp v1.4–10* (Hothorn et al., 2008), corrgram v.1.14 (Friendly, 2002) and *ggplot2 v3.0.0* (Wilkinson, 2011).

#### Differences in *Osmia bicornis* bee nest microbiome between different sample types

To compare the differences in bacterial diversity among various sample types of *O. bicornis* bee nests (larval pollen provision, soil nest enclosure, bee larvae and pupae), we assessed alpha-diversity measures such as Shannon diversity, (numbers of observed ASV) richness, and (Simpson’s) evenness. These were supplemented with alpha diversity measures of plot plant diversity and pollen diversity in the same nest chambers as used here available from Peters et al. (2022). We used an analysis of variance (ANOVA) to test for significant differences between sample types, followed by Tukey post-hoc tests (using the multcomp package) to identify specific variations. To analyze variations in the microbial composition, we calculated bacterial taxonomic beta diversity using the Bray–Curtis dissimilarity and performed permutation tests (PERMANOVAs). Non-metrical multi-dimensional scaling (NMDS) was used for visualization. We additionally examined interspecific within-group variances in microbiomes of *O. bicornis* bee nest sample types, to understand consistency or heterogeneity of microbiomes between different bee nest sample types. This involved calculating the distance of each data point from the centroid of its respective group (sample type) and conducting Kruskal-Wallis tests and Dunn tests for post-hoc analysis.

#### Effects of different bacterial acquisition pathways on *Osmia bicornis* larvae and pupae

To assess whether bacterial communities associated with bee larvae and pupae were exclusive or influenced by pollen or soil bacterial communities, we conducted the following analyses: (1) We employed Venn diagrams to visualize and compare the overlap or uniqueness of microbial taxa across sample types. This graphical approach allowed us to gain insights into the degree of similarity or dissimilarity in microbial composition between these sample types. (2) We determined the relative abundances of the 20 most abundant bacterial taxa in larval pollen provisions, soil nest enclosures, bee larvae and pupae to identify overlaps or differences in bacterial community composition across different sample types. (3) We conducted Mantel tests and partial Mantel tests using Pearson’s correlation between soil and pollen with larval and pupal communities from the same nest cells to investigate potential microbial transmission dynamics within bee nests. For partial Mantel tests, we examined three dimensions of distance matrices, comparing the bacterial compositions of bee larvae or pupae with those of pollen provisions together with soil nest enclosures.

#### Effects of land use on different sample types of *Osmia bicornis* bee nest microbiome

Lastly, we investigated the impact of land use on the microbiome of *O. bicornis* solitary bee nest sample types. Initially, we evaluated the direct impact of land use intensity, quantified by the continuous land use index (LUI) (Blüthgen et al., 2012) and separately by mowing, grazing and fertilization intensities, on the bacterial Shannon alpha diversity within bee nest sample types of *O. bicornis* (larval pollen provisions, soil nest enclosure, bee larvae and pupae) using generalized mixed-effect models (GLMMs) with Bioregion and Plot-ID as random factors. Additionally, we investigated how LUI affected the bacterial taxonomic composition using Bray–Curtis dissimilarity with NMDS for visualization purposes and permutational analysis of variance (PERMANOVA) tests based on distances of samples to centroids for each sample types.

To investigate the potential effects of land use intensity on bacterial communities across different sample types, we analyzed differences between the relative abundances of the 20 most abundant bacterial taxa in all sample types. This analysis was conducted across various land use categories, to identify overlaps or shifts in bacterial community composition associated with different land use practices. Subsequently, we performed Mantel tests (Pearson) to compare the bacterial community distances of larval pollen provisions and soil nest enclosures across different land use categories (low, intermediate, high) with the microbiome composition distances of bee larvae and pupae. Furthermore, we tested whether *O. bicornis* had comparable microbiome variance with increasing land use intensity by examining within-group (LUI) variance differences between land use categories based on the distance to the group centroid.

## Results

### Differences in *Osmia bicornis* bee nest microbiome between different nest sample types

Sequencing and bioinformatics yielded an average of 10,062 filtered reads per sample (range from 1,412 to 55,183, *SD* = 9122.183). Bacterial alpha diversity in terms of Shannon, richness and evenness varied between sample types, except the Larvae – Pupae comparison (Table 1; Supplementary Table SM3; Supplementary Figure SM2). We observed the highest ASV richness and Shannon diversity in soil nest enclosures compared to all other sample types (larval pollen provisions, larvae and pupae) (Supplementary Table SM3; Supplementary Figure SM2).

**Table 1:** Tukey’s HSD *post hoc* test results for differences in means of ASV (Shannon) diversity, (observed) richness and (Simpson’s) evenness between all *Osmia bicornis* bee nest sample types (bee larvae and pupae, larval pollen provision (=Pollen) and soil nest enclosures (=Soil)).

ASV richness				ASV Shannon				ASV evenness			
	Larvae	Pollen	Soil		Larvae	Pollen	Soil		Larvae	Pollen	Soil
Pupae	0.97	0.88	**<0.001**	Pupae	0.66	**<0.001**	**<0.001**	Pupae	0.23	**<0.001**	**0.19**
Soil	**<0.001**	**<0.01**		Soil	**<0.001**	**<0.001**		Soil	**<0.001**	**<0.001**	
Pollen	0.99			Pollen	**<0.001**			Pollen	**<0.001**		

Bold values indicate significant differences between sample types.

**Figure 1:**
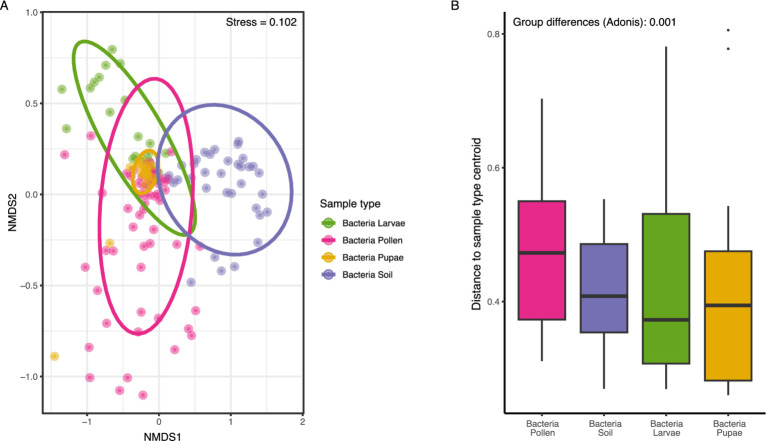
**(A)** Differences in bacterial compositions of *O. bicornis* sample types (larval pollen provisions, soil nest enclosures, bee larvae & bee pupae) (represented by different colors) by non-metrical multi-dimensional scaling (NMDS, stress = 0.102) based on Bray-Curtis distances using transformed relative abundances data of ASV (amplicon sequent variants). ASVs were plotted for all bee nest specimens for all 27 plots. Each dot represents one sample of one nest (*n* = 144) **(A)**. Variabilities in community structures of different bee species and specimen types. **(B)** The analysis of Bray-Curtis distances as distances to group centroids of each community. Differences between sample types were assessed by multivariate analysis of group dispersions (betadisper/adonis).

The bacterial community composition within the microbiome of *O. bicornis* bee nests exhibited significant differences across samples types (PERMANOVA: *F* = 9.98, df = 3, *R*^2^ = 0.17, *p <* 0.001, Figure 1A), resulting in sample type-specific microbiome compositions (Figure 1A, PERMANOVA between all sample type pairs: *p* = 0.001). Furthermore, NMDS analysis at the ASV-level revealed a higher similarity in microbiome composition between *O. bicornis* bee pupae and bee larvae compared to larval pollen provisions and soil nest enclosure microbiomes (Figure 1A). Moreover, the variability in microbiome composition, quantified as the distance to the sample type centroid, and reflecting microbiome variations within sample types, varied among sample types (Kruskal Wallis test: χ^2^ = 10.46, df = 3, *p*-value < 0.01, Figure 1B), with less variable microbiomes observed in *O. bicornis* bee pupae (distance to centroid = 0.41 ± 0.15) and soil nest enclosures (distance to centroid = 0.40 ± 0.08) compared to bee larvae (distance to centroid = 0.44 ± 0.15) and larval pollen provisions (distance to centroid = 0.47 ± 0.11) (Figure 1B). Between sample type comparisons revealed significant differences in microbiome variation between groups (Dunn-Test: bee_larvae – larval pollen provisions: *p <* 0.05, bee_pupae – larval pollen provisions: *p <* 0.01, and larval pollen provisions - soil nest enclosures: *p <* 0.01).

### Effects of different bacterial acquisition on *Osmia bicornis* larvae and pupae

We found significant overlaps in bacterial communities between larval pollen provisions and soil nest enclosures with those of bee larvae and pupae (Supplementary Figures SM3, SM4, 75 omnipresent ASVs), however also as well unique elements (pollen: 1,096, soil: 445, larvae: 314, pupae: 220 exclusive ASVs). Regarding potential bacterial acquisition pathways from soil and pollen toward bee larvae and pupae, bacterial genera, such as *Pseudomonas*, *Streptomyces*, *Acinetobacter*, *and Halomonas,* were abundant across bee larvae, pupae, and larval pollen provisions, while they were less abundant in soil nest enclosures. Higher abundances of *Bacillus* genera were found in bee larvae (19.78%) and soil nest enclosures (8.4%), contrasting with lower abundances in larval pollen provisions (3.5%) and bee pupae (1.4%) (Supplementary Figure SM4).

Pairwise comparisons between bacterial distance matrices across various sample types of *O. bicornis* nests using Mantel tests revealed a significant positive correlation exclusively between larval pollen provisions and soil nest enclosures (Table 2). Furthermore, bacterial distances between bee larvae and larval pollen provisions, including soil nest enclosures as a covariate via partial mantel tests, also showed a significant positive correlation, while no significant correlations were found for bee pupae (Table 2).

**Table 2:** Summary statistics of Mantel and Partial mantel tests of distances comparisons between bacterial compositions of different sample type (bee larvae = Larvae, bee pupae = Pupae, larval pollen provisions = Pollen and soil nest enclosures = Soil) of *Osmia bicornis* bee nests.

Test type	Sample types	Bray Curtis	
		*r*	*p*
Mantel	Larvae ~ Pollen	0.18	0.13
Mantel	Larvae ~ Soil	0.10	0.23
Mantel	Pupae ~ Pollen	0.04	0.40
Mantel	Pupae ~ Soil	0.02	0.42
Mantel	Larvae ~ Pupae	−0.18	0.78
Mantel	Pollen ~ Soil	0.27	0.01
Partial Mantel	Larvae ~ Pollen + Soil	0.31	0.05
Partial Mantel	Pupae ~ Pollen + Soil	−0.09	0.58

### Effects of land use on different sample types of *Osmia bicornis* bee nest microbiome

**Figure 2:**
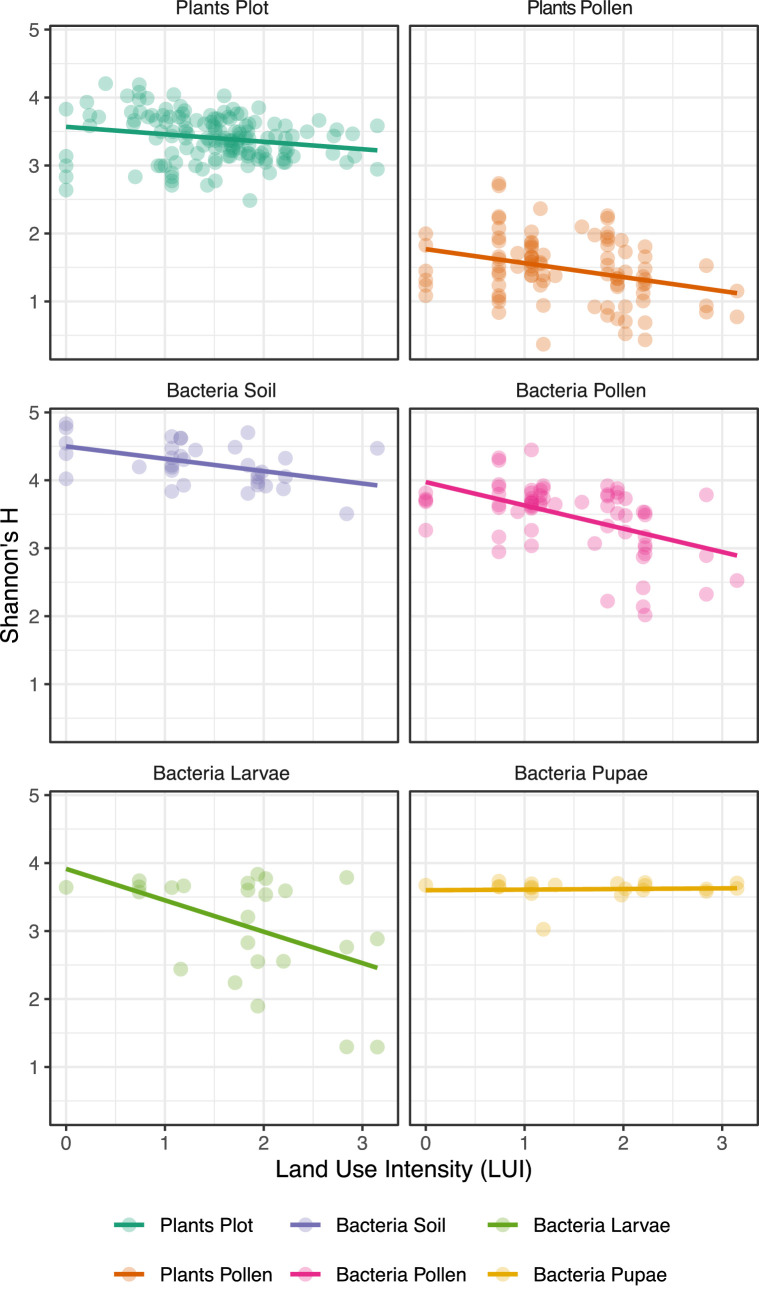
Effects of land use intensity (LUI) on **(A)** the Shannon plant diversity of plot vegetation and **(B)** pollen larval provisions, as well as Shannon bacterial diversity of **(C)** larvae, **(D)** pupae, **(E)** pollen larval provisions and **(F)** soil nest closings of *Osmia bicornis* sampled from trap nests installed at plots differing in land use intensity (LUI) in three biogeographical regions in Germany (Exploratories: Swabian Alb, Hainich-Dün and Schorfheide-Chorin). Shannon diversity is based on revealed ASVs (Amplicon sequent variants) per bee nest. Data for **(A,B)** was obtained through Peters et al. (2022) for comparison.

Within *O. bicornis* bee nests, we found negative effects of LUI on the Shannon diversity of microbiomes of pollen (*p <* 0.001), soil nest closings (*p* < 0.01) and bee larvae (*p* < 0.05), however not on pupae (Figure 2; Supplementary Figure SM5). We additionally tested for the separate effects of mowing, grazing, and fertilization intensities at plot sites, which are all integrated in the LUI index, and found significant decreases in bacterial diversity particularly with increasing mowing intensities and fertilization (Supplementary Figure SM6; Table 3). Notably, no significant impact of any land-use variable on bacterial diversity was observed for bee pupae (Figure 2). Moreover, we observed a very high relative increase in the relative abundance of *Bacillus* sp. in *O. bicornis* larvae samples and soil enclosures (Supplementary Figure SM5), respectively from low [4.25% (larvae), 4.31% (soil)] to high land use intensity field sites [37.81% (larvae), 12.02% (soil)], which represents an increase of approximately 8 times for larvae and an increase 3 times for soil enclosures.

**Table 3:** Summary statistics of individual LUI components based on generalized mixed-effect models with Bioregion and Plot-ID as random factors.

	Pollen provisions	Soil enclosures	Larvae	Pupae
Mowing	Significant decrease (*p* < 0.01)	Significant decrease (*p* < 0.05)	Significant decrease (*p* < 0.001)	No significant impact
Grazing	No significant impact	No significant impact	Significant increase (*p* < 0.01)	No significant impact
Fertilization	Significant decrease (*p* < 0.01)	Significant decrease (*p* < 0.01)	Significant decrease (*p* < 0.01)	No significant impact

**Figure 3:**
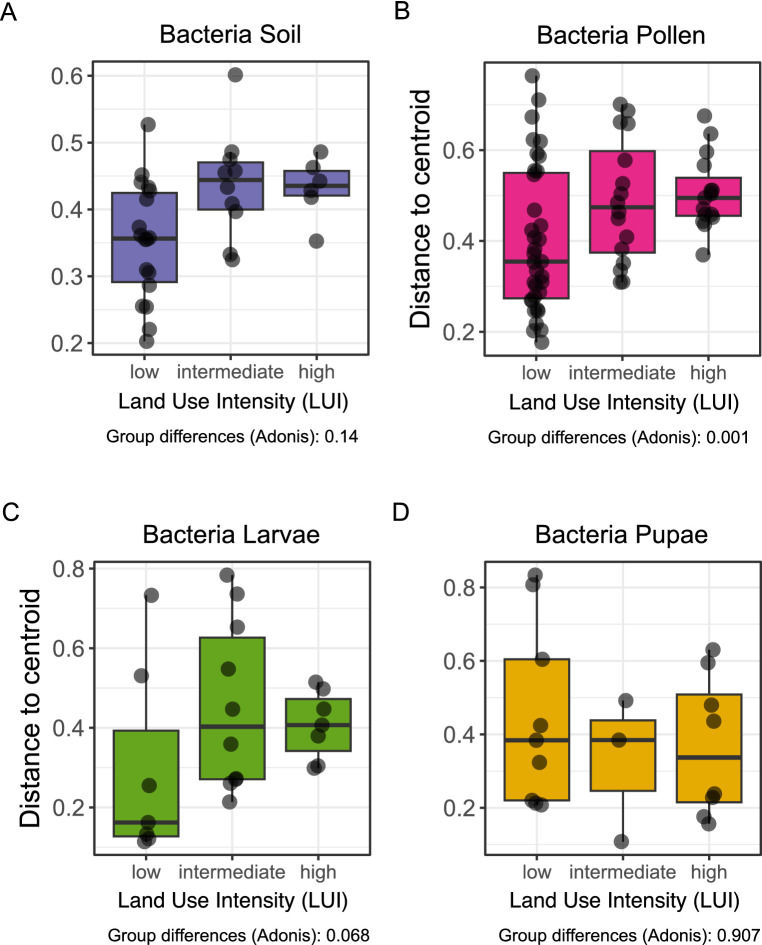
Variabilities in community structures of different land-use intensities (LUI categories: low-high) and *O. bicornis* bacterial microbiomes. The analysis of Bray-Curtis distances represents the beta-diversity as distances to group centroids of each community. Differences between land-use categories within a specimen group **(A–D)** were assessed by multivariate analysis of group dispersions (betadisper/adonis).

Bray-Curtis dissimilarities (multivariate homogeneity of group dispersions, *R*^2^ = 0.15, *p* < 0.001) showed significant changes in the variability of bacterial communities in pollen provisions, increasing from low (distance to centroid = 0.40 ± 0.11), intermediate (0.48 ± 0.11) to high intensity field sites (0.50 ± 0.09) (Figure 3). Variability in *O. bicornis* bacterial larval communities also tended to increase (*R*^2^ = 0.18, *p* = 0.068) from low to high intensity field sites (distance to centroid; low: 0.29 ± 0.09, intermediate: 0.45 ± 0.08, high: 0.41 ± 0.12) (Figure 3). We observe neither for soil enclosures nor pupae bacterial communities significant variability differences between low and high land use intensities (multivariate homogeneity of group dispersions, soil: *R*^2^ = 0.13, *p =* 0.14, pupae: *R*^2^ = 0.18, *p =* 0.91).

## Discussion

Microbiomes of Megachilid solitary bees have previously been reported to be mostly distinct and species-specific, but also highly variable on an individual scale depending on environmental factors, transmission routes or bee developmental stages (Gilliam et al., 1990; Inglis et al., 1998; Potts et al., 2005; Mohr and Tebbe, 2006; Keller et al., 2013; McFrederick et al., 2017; McFrederick and Rehan, 2019; Keller et al., 2021). In this study, we investigated the bacterial diversity and composition in the Megachilid trap-nesting polylectic solitary bee *Osmia bicornis* at different developmental stages (larvae, pupae) and in different nesting compartments (larval pollen provisions and soil nest enclosure). The latter two were considered as potential bacterial acquisition pathways. Finally, we assessed how land use intensity, management practices (mowing, grazing and fertilization), as well as flower availability influenced bacterial composition and diversity of bee and nest samples.

### Differences in *Osmia bicornis* bee nest microbiome between different nest sample types

When assessing microbial richness and diversity across various *O. bicornis* sample types, we observed significant differences in bacterial community composition across sample types, suggesting a sample type-specific microbiome of *O. bicornis* bee nests which aligns with previous studies (Voulgari-Kokota et al., 2018). Moreover, we could show that soil samples, used for the segregation of individual nest chambers, demonstrated the highest bacterial diversity among all the examined nest materials (Keller et al., 2013; Voulgari-Kokota et al., 2020). This is in contrast to honey bee nest walls, which are built of waxes and propolis as a protective measure against microbial colonization and proliferation, and show little bacterial diversity (Anderson et al., 2011). The high soil-derived bacterial diversity in *O. bicornis* nests contrasts the antimicrobial environments typically found in managed hives where honey bee pupae develop (Gilliam, 1971).

### Effects of different bacterial acquisition on *Osmia bicornis* larvae and pupae

Bacterial communities within solitary bee nests can be influenced by pollen used to provision solitary bee larvae (McFrederick et al., 2017) and thus the spectrum of allocated plant sources (Voulgari-Kokota et al., 2019a; Keller et al., 2021). We also found ASV overlaps and positive correlations between the bacterial communities between the pollen and soil microbiomes with those of the larvae. This suggests that environmentally introduced microbiomes from both pollen and soil influenced the bacterial communities of our larvae (Voulgari-Kokota et al., 2018; Cohen et al., 2020; Steffan et al., 2024). Certain bacterial genera, such as *Pseudomonas, Streptomyces, Acinetobacter*, and *Halomonas*, were shared between and enriched in bee larvae, pupae, and larval pollen provisions, while *Acidobacteria* and *Bacillus* exhibited higher abundances in soil nest enclosures and larvae as also reported in other studies (Keller et al., 2013; Lozo et al., 2015; McFrederick and Rehan, 2016; Voulgari-Kokota et al., 2018; Fernandez De Landa et al., 2023). While the role of *Pseudomonas* for bees is not quite understood (Fernandez De Landa et al., 2023), it is known for its diversity and prevalence on plant surfaces and in floral microbiomes (Roberson and Firestone, 1992; Chang et al., 2007; Voulgari-Kokota et al., 2018; Gaube et al., 2020; Steffan et al., 2024). Similarly, Acinetobacter is commonly found in nectar and associated with wild bees and has been shown to contribute to pollen germination and nutrient uptake within the protoplasm (Alvarez-Pérez et al., 2012; Fridman et al., 2012; Christensen et al., 2024). Soil poses an potential alternative bacterial transmission pathway, as shown by other studies for mud/soil nest enclosures (Keller et al., 2018; Voulgari-Kokota et al., 2020) or cut leaves (Rothman et al., 2019). Bacterial hubs or reservoirs are especially relevant during the early stages of bee development (Keller et al., 2018; McFrederick and Rehan, 2019; Dew et al., 2020; Christensen et al., 2023). For example, the bacterial community of pollen/nectar provisions in *O. cornifrons* brood cells initially exhibits a diverse bacterial composition, which is gradually reduced and altered over time by larval feeding (Kueneman et al., 2023). This process involves the suppression or elimination of less common taxa, while bacterial endosymbionts typically associated with insects and a range of plant pathogens proliferate (Kueneman et al., 2023).

Despite the observed overlaps with environmental materials, we still observed that the microbiomes of bee pupae and larvae were more similar to each other than to those from larval pollen provisions and soil nest enclosures. The overlap between pupae and larvae indicates a strong potential for bacterial proliferation and transmission from before to after metamorphosis (Voulgari-Kokota et al., 2019b). Bee pupae (and soil nest) enclosures exhibited lower variability compared to bee larvae and larval pollen provisions, indicating greater microbiome stability in these sample types. Furthermore, pupal microbiomes did not correlate with such of pollen nor soil in their composition as a potential result of environmental bacteria reduction over time in the nest (Kueneman et al., 2023). This suggests that while transgenerational passthrough is managed by some bacteria, there is likely a potential filter in the transfer of the microbiome from larvae to pupae. How emerging solitary bees recover their microbiome remains unclear. Proposed routes include inoculation by chewing through remaining nest materials or from flower hubs (Keller et al., 2021), which can however be excluded here as pupae did not emerge yet. Unlike social bees, solitary bee species lack adult nursing, which aids in establishing stable transgenerational microbial communities (Turnbaugh and Gordon, 2009; Danforth et al., 2019). Rather than maintaining a similarly consistent or conserved microbiome across individuals, adult solitary bees appear to exhibit microbial compositions that are more influenced by the environments and collected materials of previous generations, in line with our results here (McFrederick et al., 2012; McFrederick et al., 2017; Voulgari-Kokota et al., 2020; Kapheim et al., 2021; Nguyen and Rehan, 2023). Interestingly, a recent investigation conducted within the brood cells of the solitary bee *Anthophora bomboides* also revealed the presence of individually consistent microbiomes persisted throughout multiple life stages, suggesting a certain degree of individual microbiome stability (Christensen et al., 2023).

### Effects of land use on different sample types of *Osmia bicornis* bee nest microbiome

**Figure 4:**
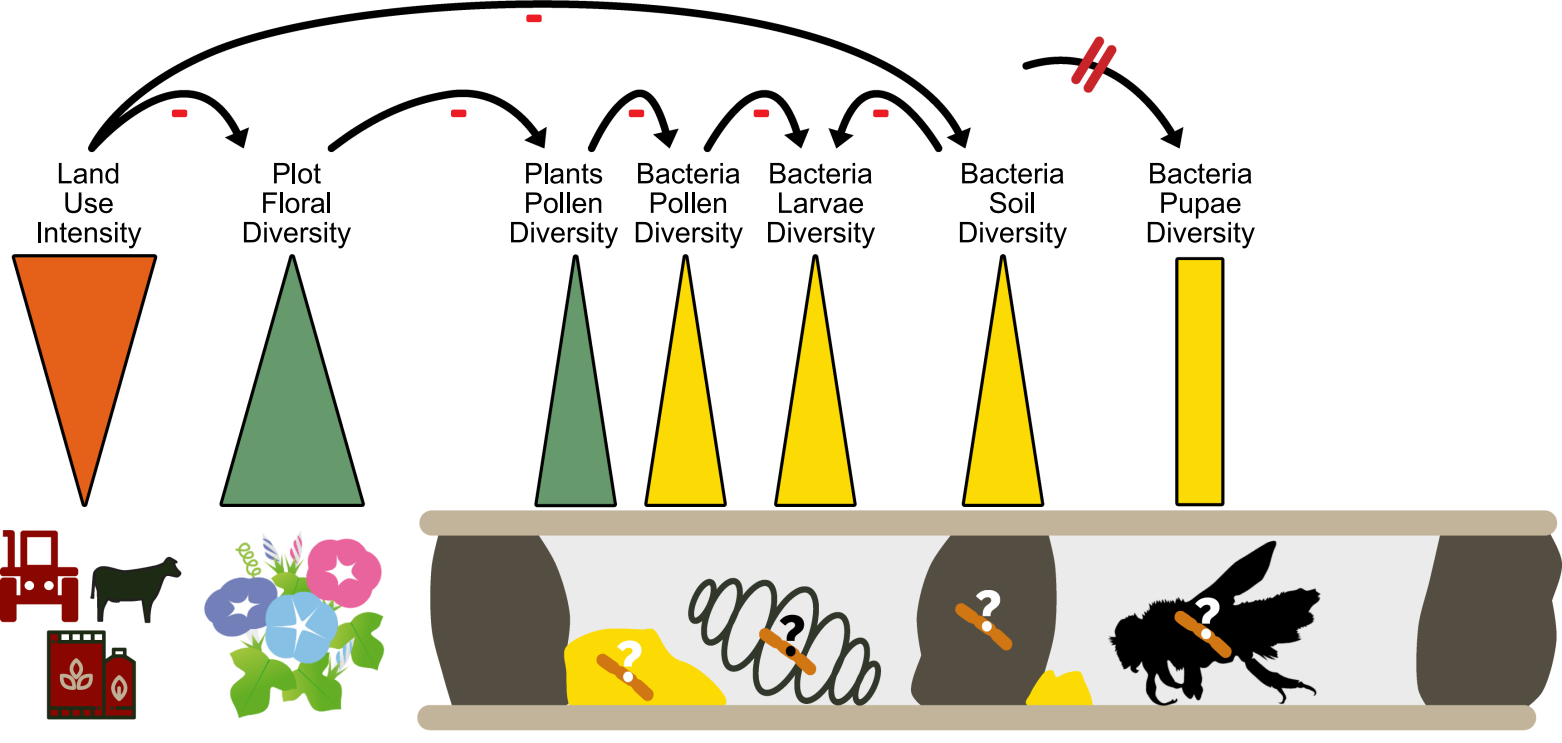
Schematic overview about the influence of land use intensity on bacterial microbiome diversity of different bee nest components, especially bee larvae.

We found strong negative effects of land use intensity (LUI) on the bacterial diversity of larval pollen provisions, bee larvae and soil nest enclosures, more in detail with increasing mowing and fertilization intensities, but not on the Shannon bacterial diversity nor composition of bee pupae. The negative correlation is likely an indirect effect, since the diversity of flowering plant species on plots also correlated positively with bacterial Shannon diversity of larval pollen provision, soil nest enclosures and bee larvae. The direct link here is likely between the available spectrum of flowering plants and the microbiome associated with *O. bicornis* bee nests. A study conducted by Nguyen and Rehan (2022) demonstrated that microbial composition in the small carpenter bee, *Ceratina calcarata*, varies across different urban land use gradients. Specifically, microbes like *Acinetobacter* and *Apilactobacillus* are more common in less urbanized areas, while the fungus *Penicillium* is more prevalent in developed urban areas. Interestingly, in the study of Fernandez De Landa et al. (2023), no connection was found between the overall gut microbiome composition and land use intensity for the solitary bees *Xylocopa augusti*, *Eucera fervens*, and *Lasioglossum*. However, changes were observed for the bacterial symbionts *Snodgrassella* and *Nocardioides*, which displayed higher abundances in less anthropogenically impacted sites. Similarly, higher land use intensity also led to flower bacterial communities that were less phylogenetically diverse and more uniform in composition, and to a reduced floral bacterial species pool at high land-use intensity plots (Gaube et al., 2020). This supports the idea that LUI indirectly impacts the microbiome associated with pollen collected by solitary bees and consequently bee offspring via the food and nest resources allocated in nests (Figure 4).

Moreover, we found the variability and heterogeneity of bacterial microbiomes in larval pollen provisions and bee larvae to increase with land use intensification, which might indicate more erratic microbiomes or alternative floral sources used in intensively used areas. The stage-specific vulnerability of developing bees to environmental stressors (Ferguson et al., 2018; Christensen et al., 2024) combined with the lack of microbiome consistency in bee larvae growing up in areas of differing land use intensity (Engel et al., 2016) might increase the risk of the bees’ microbiome being more susceptible to and thus invaded by pathogenic environmental bacteria (Voulgari-Kokota et al., 2020; Fernandez De Landa et al., 2023). Interestingly, we also observed higher variability in the bacterial compositions of soil nest enclosures in high and intermediate than low land use intensity sites, which have been described as barriers for pathogen spillover in solitary bee nests (Voulgari-Kokota et al., 2020). In fact, certain bacteria, e.g., *Bacillus* sp., were present in higher abundances in *O. bicornis* bee larvae and soil nest closings at highly intensified areas. This genus was the major suspect in causing mortality in *O. bicornis* larvae in the study of Voulgari-Kokota et al. (2020). These findings underscore the necessity for in-depth investigations of microbial acquisition routes of certain bacterial genera of the distinct developmental stages of various bee species over time (Weinhold et al., 2024). Interestingly, land use intensity did not affect the bacterial composition of *O. bicornis* bee pupae. This could mean that either such bacteria cannot pass transgenerational filters or that only those larvae that are capable of maintaining or establishing a healthy microbiome, despite external influences, can develop properly and survive metamorphosis (Voulgari-Kokota et al., 2020; Brar et al., 2023). This may also explain (at least partially) why the number of vital larvae and nesting cells of *O. bicornis* decreased with increasing land use intensity in a previous study at the same sites (Peters et al., 2022).

As proposed here, land use indirectly affects pollen bacterial communities via alterations in plant spectra and collections of environmental microbes via pollen provisions (Leonhardt et al., 2022; Peters et al., 2022). The resulting decrease in bacterial diversity and shifts in community composition can directly affect the invasion potential of pathogenic bacteria or indirectly the larvae’s ability to effectively uptake nutrients. Thus, microbiome shifts at high land use intensity likely result in less healthy larvae and increasing larval mortality. This highlights the complex interaction between land use practices, microbial communities, and bee health, underscoring the importance of considering microbiome dynamics in agriculturally managed landscapes. Untangeling these connections can in turn provide deeper insights into potential mitigation strategies to reduce the negative impacts of agricultural intensification on pollinator populations and ecosystem health.

## Conclusion

Human activities and land use intensity contribute to solitary bee population declines, shifts in wild bee community compositions, and adverse impacts on bee species and health. These effects may be mediated or enhanced by changes in the structure, composition, and diversity of bee microbiomes. Our research highlights the complex interplay of direct and indirect environmental factors shaping solitary bee microbiomes, emphasizing the need for further investigation into the functions of bacterial communities. Specifically, our study revealed that the microbiome of *Osmia bicornis* shows sample type-specific variations influenced by environmental conditions, transmission pathways, and developmental stages. We also observed that increasing land use intensity significantly decreased bacterial diversity in larval pollen provisions, soil nest enclosures, and larval microbiota, while increasing the abundance of potentially pathogenic *Bacillus* spp. in bee larvae and soil nest enclosures. These findings underscore the urgency of researching the multifaceted environmental stressors affecting solitary bee microbiomes to enhance our understanding of bee health and ecosystem stability.

## Data Availability

Sequencing data of bacterial composition based on 16S metabarcoding is deposited at the NCBI sequence read archive (https://www.ncbi.nlm.nih.gov) under BioProject: PRJNA763907. The following data sets are available at BEXIS (https://www.bexis.uni-jena.de): Dataset IDs 23586 and 24247 Vegetation Records for 150 Grassland EPs (2008–2018). Dataset IDs 25086 and 31514 data on management and land use intensity and Dataset IDs 27229 Plant species diversity based on rDNA gene sequences (ITS2) of trap nesting solitary bee species 2017–2018.
