# Supplementary material for: Direct and indirect effects of land use on microbiomes of trap-nesting solitary bee larvae and nests
Source: Front Microbiol. 2025 Jan 9;15:1513096. doi: 10.3389/fmicb.2024.1513096 (PMC11753253; doi:10.3389/fmicb.2024.1513096)

**Supplementary material**

Direct and indirect effects of land use on microbiomes of trap-nesting solitary bee larvae and nests

Birte Peters^1,2,3^, Sara Diana Leonhardt^4^, Michael Schloter^5^, Alexander Keller^2,6^

^1^ Department for Animal Ecology and Tropical Biology, Biocenter, University of Würzburg, Am Hubland, 97074 Würzburg, Germany

^2^ Center for Computational and Theoretical Biology, University of Würzburg, Emil Fischer Straße, Hubland

^3^ Department of Biodiversity and People, Helmholtz Center Leipzig, German Centre for Integrative Biodiversity Research (iDiv), 04103 Leipzig

^4^ Department of Ecology and Ecosystem Management, Technical University of Munich, 85354 Freising, Germany

^5^ Environmental Genomics, Helmholtz Centrum Munich, Technical University of Munich, 85764 Munich, Germany

^6^ Cellular and Organismic Networks, Faculty of Biology, Ludwig-Maximilians-Universität Munich, 82152 Planegg-Martinsried, Germany, Email: [keller@bio.lmu.de](mailto:keller@bio.lmu.de), Phone: +49 931 3184427

**Tables**

**Table SM1:** Incorporated plots (Plot_ID) from the three Biodiversity Exploratories – Schorfheide-Chorin (SEG), Hainich-Dün (HEG), and Swabian Alb (AEG) within our research. Each plot is assigned a land-use intensity index (LUI) value derived from grazing (G), mowing (M), and fertilization (F) activities.

| **Plot_ID** | **G** | **M** | **F** | **LUI** |
| --- | --- | --- | --- | --- |
| AEG1 | 0 | 1.91 | 2.34 | 2.06 |
| AEG2 | 0 | 2.87 | 9.31 | 3.49 |
| AEG3 | 0 | 1.91 | 0 | 1.38 |
| AEG4 | 1.24 | 0.96 | 1.6 | 1.95 |
| AEG5 | 1.01 | 0.96 | 1.6 | 1.89 |
| AEG6 | 0.76 | 0.96 | 0.81 | 1.59 |
| AEG7 | 0.34 | 0 | 0 | 0.59 |
| AEG8 | 0.4 | 0.96 | 0 | 1.16 |
| AEG9 | 0.82 | 0 | 0 | 0.9 |
| HEG1 | 0 | 1.91 | 10.24 | 3.49 |
| HEG2 | 0.2 | 1.91 | 3.24 | 2.31 |
| HEG3 | 0.2 | 1.91 | 3.24 | 2.31 |
| HEG4 | 0 | 0.96 | 3.15 | 2.03 |
| HEG5 | 0 | 1.91 | 2.46 | 2.09 |
| HEG6 | 0.17 | 0.96 | 4.88 | 2.45 |
| HEG7 | 5.13 | 0 | 0 | 2.26 |
| HEG8 | 1.48 | 0 | 0 | 1.22 |
| HEG9 | 0.58 | 0 | 0 | 0.76 |
| SEG1 | 0 | 0.96 | 0 | 0.98 |
| SEG2 | 1.17 | 0 | 0 | 1.08 |
| SEG3 | 1.23 | 0.96 | 0 | 1.48 |
| SEG4 | 0 | 0 | 0 | 0 |
| SEG5 | 0 | 0.96 | 0 | 0.98 |
| SEG6 | 3.04 | 0.96 | 0 | 2 |
| SEG7 | 2.32 | 0 | 0 | 1.52 |
| SEG8 | 3.76 | 0.96 | 0 | 2.17 |
| SEG9 | 2.39 | 0 | 0 | 1.55 |

**Table SM2:** Information about sample size (N) of *Osmia bicornis* bee specimens (bee larvae & pupae) and nesting material like larval pollen provisions (pollen) and soil nest enclosures (soil) using 16S metabarcoding.

| **Bee species** | Nesting material | | Bee | |
| --- | --- | --- | --- | --- |
| *Osmia bicornis* | Pollen (N) | Soil (N) | Larvae (N) | Pupae (N) |
|  | 66 | 34 | 24 | 20 |

**Table SM3:** Richness, diversity, and evenness of the microbiome of bee larvae, pupae, larval pollen provisions (pollen), and soil nest enclosures of *O. bicornis* bee nests. The given values represent means and their respective standard deviations (SD).

| *Osmia bicornis* |  | Richness | Diversity | Evenness |
| --- | --- | --- | --- | --- |
|  | Larvae | 416.95±146.24 | 3.13±0.76 | 0.52±0.12 |
|  | Pupae | 368.11±136.53 | 3.46±0.33 | 0.60±0.08 |
|  | Pollen | 434.37±193.99 | 2.27±0.79 | 0.38±0.13 |
|  | Soil | 1418.03±450.73 | 5.01±0.93 | 0.69±0.11 |

**Figures**

**Figure SM1:** Artificial trap-nests, specifically designed to attract cavity-nesting solitary bee species, were installed at the wooden fence of the weather station in each plot (left) across the three Biodiversity Exploratories (right). Each trap nest was equipped with a plastic tube containing 60-80 hollow reed sticks, measuring approximately 20 cm long and 4-12 mm in width (middle). Monitoring of trap nests occurred at 8-10 week intervals from March to October to encompass the full flight period of trap-nesting solitary bees. Sample collection involved five sessions in 2017 and three sessions in 2018. Reed sticks found to have sealed entrances were replaced with fresh sticks and promptly transported to the University of Würzburg laboratory. The research plots were distributed geographically and situated in three different locations in Germany: the UNESCO Biosphere Reserve Swabian Alb (ALB) in southern Germany, the National Park Hainich-Dün (HAI) in central Germany, and the UNESCO Biosphere Reserve Schorfheide-Chorin (SCH) in the northeast of Germany.Bee collection activities were conducted under permits (ALB: AZ: 55-8/8848.02-07, HAI: AZ: 63.02/15.02.11-bio_expl2017.2 & AZ: 1011-17-301, SCH:AZ: 4743/128+5#69122/2018). Additionally, a schematic map depicting the layout of the Biodiversity Exploratory framework is provided for reference (right).

**
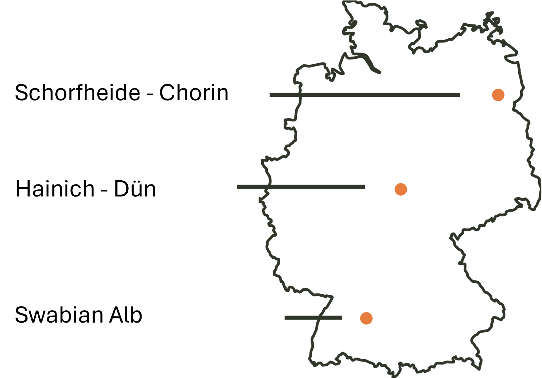
**
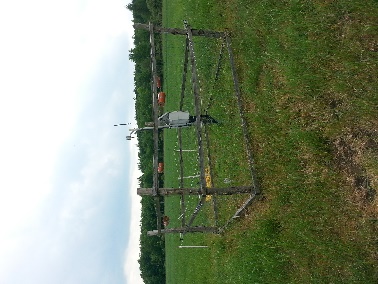

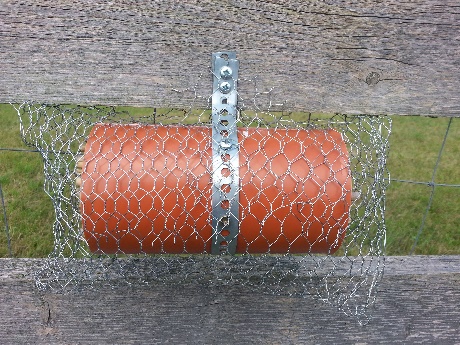


**Figure SM2:**  Richness (left) and Shannon diversity (right) of microbial communities in different *O. bicornis* sample types (larval pollen provision, soil nest enclosure, larvae, and pupae) based on the number of ASVs.
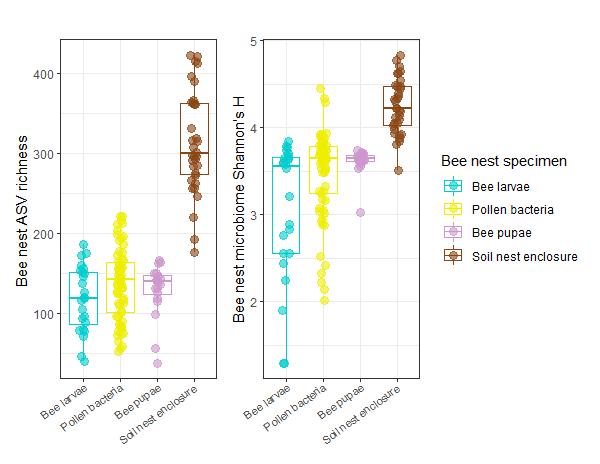


**Figure SM3:** Venn diagrams analysis of shared amplicon sequence variants (ASVs) among the twenty most abundant taxa across all investigated sample types of *O. bicornis* bee nests. The input ASV table represents presence/absence data, with occurrences of shared presence across bacterial communities of the different sample types. Specifically, 75 ASVs were found to be shared among all four sample types, including larval pollen provisions, bee larvae, bee pupae, and soil nest enclosures.

**
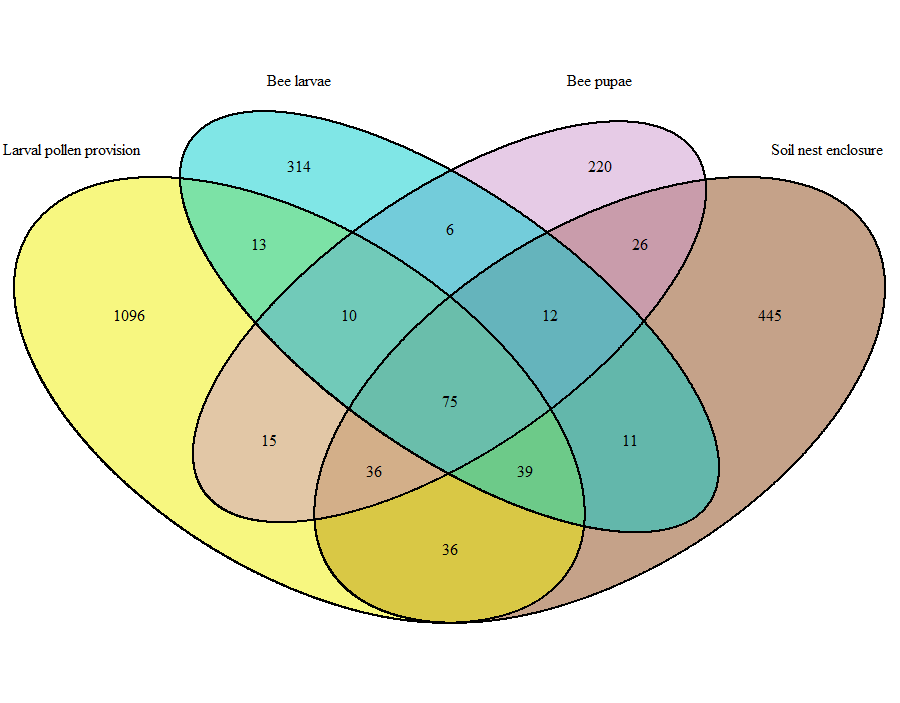
**

**Figure SM4:**Mean relative abundance of bacterial taxa (twenty most abundant taxa) of *O. bicornis* bee nest samples (larval pollen provision (N=66), soil nest enclosure (N=34), larvae (N=24), pupae (N=20)).

**
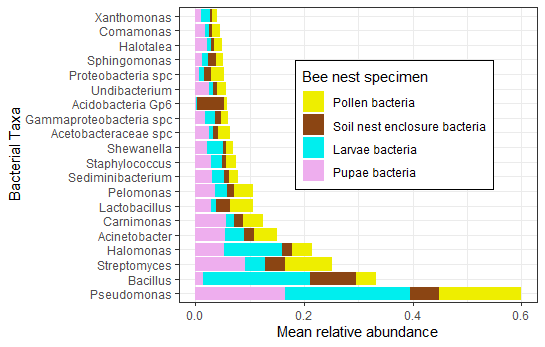
**

**Figure SM5:**Mean relative abundance of bacterial taxa (twenty most abundant taxa) of *O. bicornis* bee nest samples (larval pollen provision (=pollen), soil nest enclosure (=soil), larvae and pupae). according to different land-use intensities (Low:LUI<1.1, Intermediate:LUI 1.11-2.3,High:LUI>2.31)

**
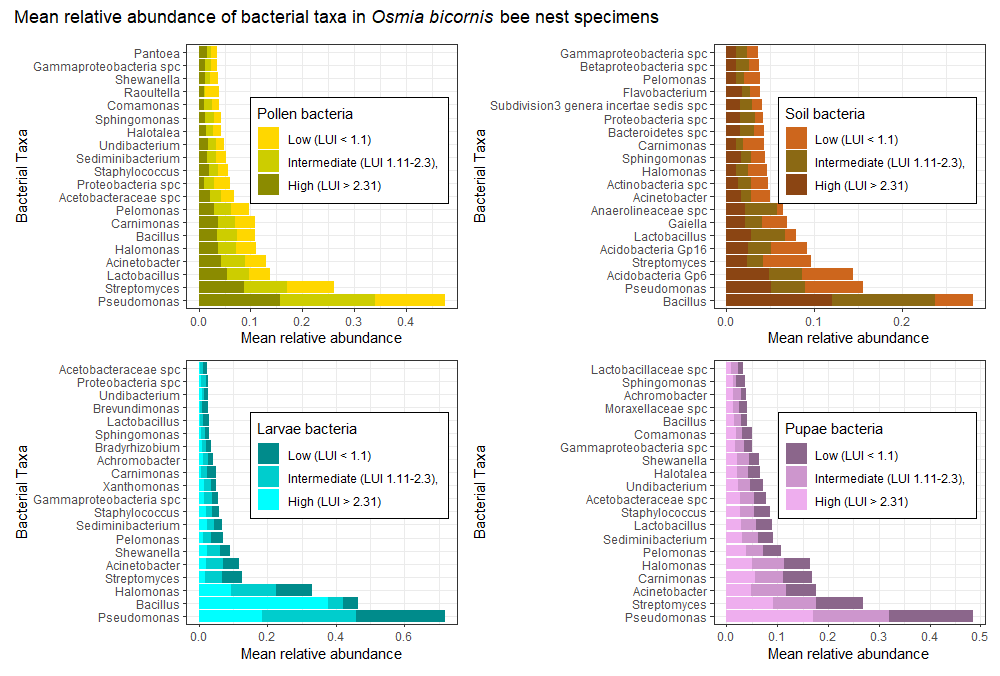
**

**Figure SM6:** Effects of Mowing (A), Grazing (B) and Fertilization (C) intensity on Shannon bacterial diversity of bee larvae &pupae, larval pollen provisions and soil nest enclosure of *O. bicornis* sampled from trap nests installed at plots differing in land-use intensity (LUI) in three biogeographical regions in Germany (Exploratories: Swabian Alb, Hainich-Dün and Schorfheide-Chorin). Shannon diversity is based on revealed ASVs (Amplicon sequent variants) per bee nest.


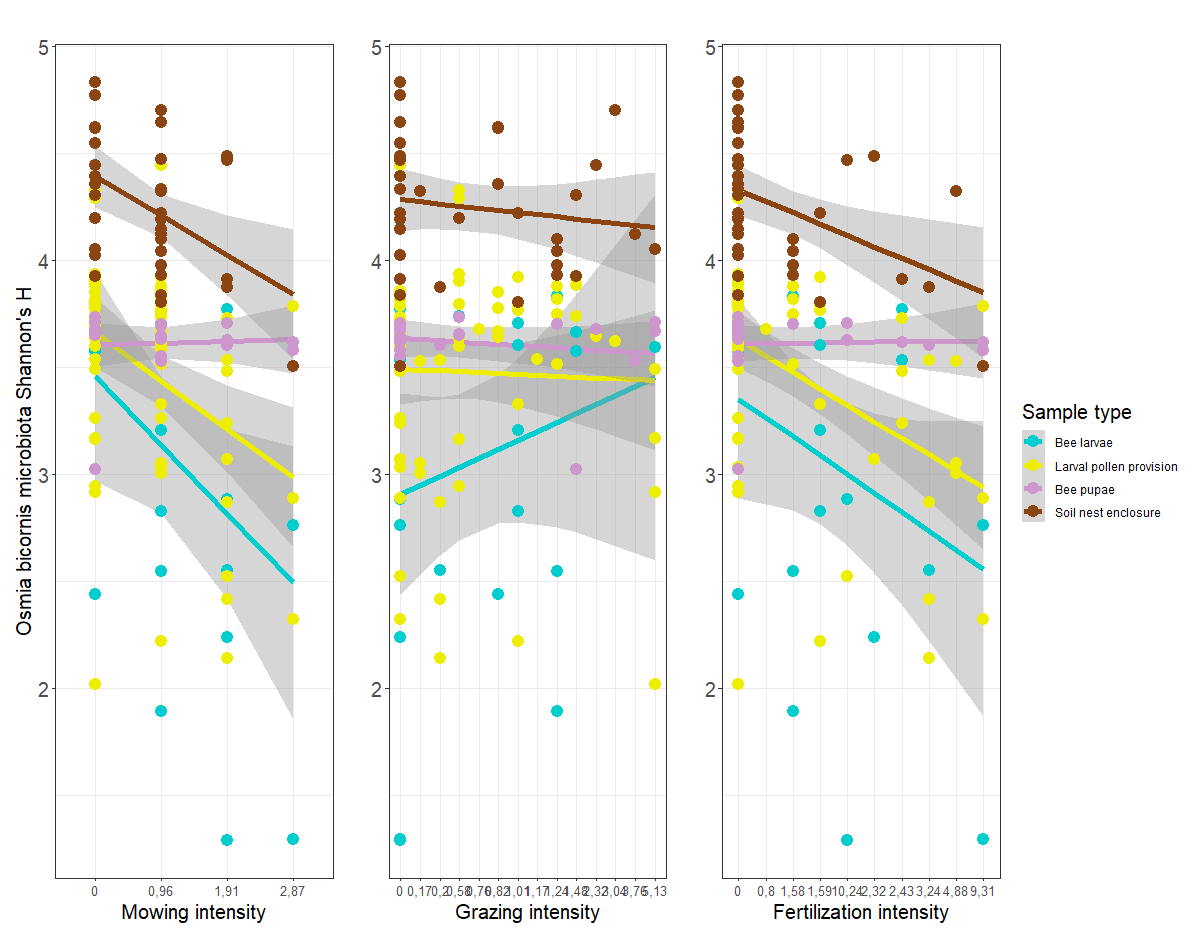

Supplement: Supplementary file 1 [file Data_Sheet_1.docx]
